# Functions of human olfactory mucus and age-dependent changes

**DOI:** 10.1038/s41598-023-27937-1

**Published:** 2023-01-18

**Authors:** Tomohiro Shirai, Dan Takase, Junkichi Yokoyama, Kuniyuki Nakanishi, Chisaki Uehara, Naoko Saito, Aya Kato-Namba, Keiichi Yoshikawa

**Affiliations:** 1 Sensory Science Research, Kao Corporation, 2606 Akabane, Ichikai-machi, Haga, Tochigi Japan; 2 Analytical Science Research, Kao Corporation, 1334 Minato, Wakayama, Wakayama Japan; 3 Department of Otolaryngology-Head and Neck Surgery, Edogawa Hospital, 2-24-18 Higashikoiwa, Edogawa, Tokyo Japan; 4 Present Address: Department of Otolaryngology-Head and Neck Surgery, Nadogaya Hospital, 2-1-1 Shinkashiwa, Kashiwa, Chiba Japan

**Keywords:** Olfactory system, Olfactory receptors, Ageing

## Abstract

Odorants are detected by olfactory sensory neurons, which are covered by olfactory mucus. Despite the existence of studies on olfactory mucus, its constituents, functions, and interindividual variability remain poorly understood. Here, we describe a human study that combined the collection of olfactory mucus and olfactory psychophysical tests. Our analyses revealed that olfactory mucus contains high concentrations of solutes, such as total proteins, inorganic elements, and molecules for xenobiotic metabolism. The high concentrations result in a capacity to capture or metabolize a specific repertoire of odorants. We provide evidence that odorant metabolism modifies our sense of smell. Finally, the amount of olfactory mucus decreases in an age-dependent manner. A follow-up experiment recapitulated the importance of the amount of mucus in the sensitive detection of odorants by their receptors. These findings provide a comprehensive picture of the molecular processes in olfactory mucus and propose a potential cause of olfactory decline.

## Introduction

Information on odorants is used to detect dangers, select and taste food, and recognize as well as communicate with other individuals. A decreased olfactory ability increases the risk of personal danger and is associated with decreased appetite, physical and mental issues and, ultimately, a lower quality of life (QOL)^1,2^. The importance of olfaction has been further highlighted by the coronavirus disease 2019 pandemic, as the condition frequently causes olfactory dysfunction^3^. Olfactory function commonly declines with aging and with the development of neurodegenerative disorders such as Alzheimer’s disease^4^. Cases of age-related decline in olfaction have also spiked in our increasingly aging society. Understanding the molecular mechanisms underlying olfactory dysfunction is required to develop an effective treatment strategy.

Inhaled odorants reach the olfactory cleft (OC) at the top of the nasal cavity. Olfactory sensory neurons (OSNs) scattered in the OC detect odorants using approximately 400 olfactory receptors (ORs) and convey odor information to higher brain areas via the olfactory bulb^5^. In addition, emerging evidence suggests that an important process likely occurs before odorants are recognized by ORs. The OC of mammals is covered by a thin layer of olfactory mucus secreted by Bowman’s glands and sustentacular cells^6^. Olfactory mucus contains various components including odorant-binding proteins (OBPs), metabolic enzymes, and bioinorganic elements^7–21^. Owing to the capacity of these components to interact with odorants, they play important roles in efficient olfaction in humans by transporting volatiles to the odorant-binding sites of ORs or through the enzymatic metabolism of odorants into nontoxic structures and/or structures with higher affinity for ORs. A continuous flow of mucus may ensure efficient washing of the mucosa and the steady elimination of odorants, which constitutes a critical step in recovering sensitivity following odorant exposure^22^. Direct evidence for the functions of the olfactory mucus and their importance in perception is rare owing to the difficulty in sampling enough pure olfactory mucus for analyses. Some studies have obtained olfactory mucus as a saline solution from an irrigated nasal cavity, causing dilution and including impurities from the surrounding regions^7,8^. Although others have directly collected pure olfactory mucus samples from human participants, they have reported only limited insights into its functions^9–11,13,14^.

Aging-related olfactory decline is widespread in the population aged ≥ 65 years, with no established treatment. This decline has been reported in several aspects of olfactory skills, including sensitivity, discrimination, identification, and recovery from adaptation^23–26^. Some potential causes of aged olfaction have been proposed^27^. Age-dependent degeneration of the neuroepithelium, including basal cell abnormalities, has been consistently observed in animal models as well as in humans^28–32^. This degeneration leads to a decreased number of OSNs and causes atrophic changes in the olfactory bulb^33,34^. A previous study suggested age-related changes in the expression level of ORs^35^, although another study indicated that they had a stable expression pattern during aging^36^. Changes in higher brain regions are involved in age-related functional deficits^37^. However, the contribution of these changes to olfactory decline remains unclear. We conducted an association study between age-related decline in olfactory sensitivity and the olfactory mucus proteome^10^. Although several proteins were found to be associated with olfactory sensitivity, none appeared to be involved directly in the detection of odorants to which the elderly have decreased sensitivity. Thus, the major cause of age-related deficits in olfaction remains undetermined.

This study clarified the functions of human olfactory mucus and their age-dependent changes. We conducted a study that combined the collection of pure olfactory mucus with olfactory tests. We also conducted follow-up in vitro and in vivo experiments to explain the observed functional associations.

## Results

### Individually varied amounts of olfactory mucus

**Figure 1:**
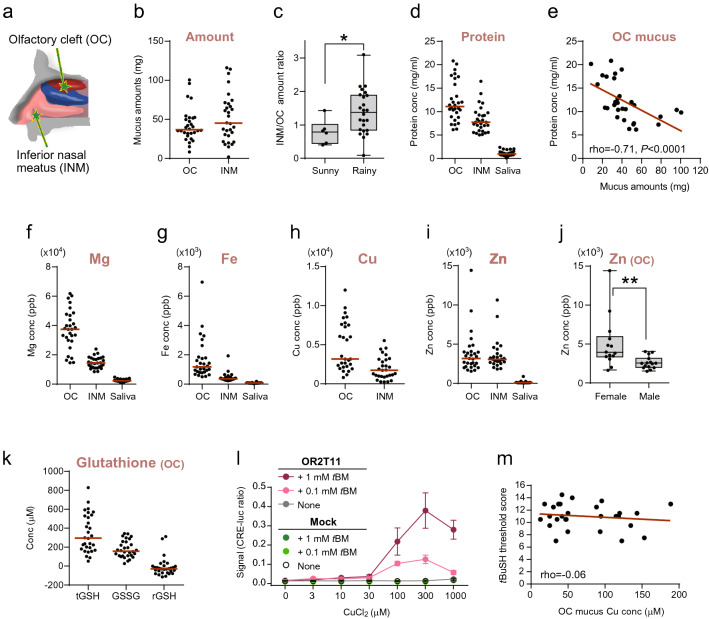
Basic properties and individual variability of olfactory mucus. (**a**) Schematic drawing of the human nasal cavity showing the location of mucus sampled in this study. Mucus samples were collected from the olfactory cleft (OC) and inferior nasal meatus (INM). (**b**) Distribution and median value (red horizonal line) of nasal mucus amount collected from 30 participants. Each dot represents the average amount of mucus from the left and right nostrils of a subject. (**c**) Weather dependency of the amount of INM mucus collected. The amount of INM mucus was normalized to the amount of OC mucus. The whisker plots show the median, first and third quartiles, and upper and lower limits in the rainy weather (n = 6) and sunny weather (n = 24) groups. (**d**) Distribution of total protein concentration in the OC mucus (n = 30), INM mucus (n = 29), and saliva (n = 30). (**e**) Correlation between the amount and protein concentration in the OC mucus (n = 30). (**f–i**) Distribution of Mg, Fe, Cu, and Zn concentrations in the OC mucus (n = 29), INM mucus (n = 29), and saliva (n = 30). Cu ion was not detected in the saliva. (**j**) Female-specific enrichment of Zn in the OC mucus. Zn concentration in the mucus of 14 female and 15 male participants are plotted. (**k**) Concentration of total glutathione (tGSH), oxidized glutathione (GSSG), and reduced glutathione (rGSH) in the OC mucus (n = 30). (**l**) Dose–response analysis of the activation of OR2T11-expressing HEK293T cells against 0.1 mM or 1 mM of *tert*-butyl mercaptan (*t*BM) with increasing concentrations of CuCl_2_ supplemented in the cell culture medium. Data are shown as the mean ± SE from three independent experiments. Cells transfected without OR2T11 were also assayed (mock). (**m**) No significant association between Cu concentration in the OC mucus and olfactory threshold score was found (n = 28 participants). Spearman’s correlation was also not statistically significant (*P* > 0.05). Significance was assessed with a Mann–Whitney U-test. *, *P* = 0.0255; **, *P* = 0.0043.

There is a lack of description of the basic properties and functions of pure olfactory mucus in humans. We collected olfactory mucus directly from 30 healthy participants without sensory complaints aged 20–67 years. Following the instillation of topical lidocaine, a neurosurgical pad was placed in the olfactory groove between the middle turbinate and superior nasal septum (OC, Fig. 1a) and between the inferior turbinate and inferior nasal septum (INM) under direct visualization using an endoscope. After 5 min, the olfactory mucus absorbed by the pads was collected and subjected to subsequent analyses.

The amount of collected mucus samples differed considerably between participants and did not follow a normal distribution (*P* < 0.01, Shapiro–Wilk normality test, Fig. 1b, Supplementary Table 1). The weight of the OC mucus samples ranged from 7 to 144 mg (median, 36.9 mg) per nasal cavity in 30 participants, whereas that of the INM mucus ranged from 0 to 135 mg (median, 45.2 mg). These variations were not caused by technical issues because the amounts of OC mucus collected from each side of the nostril through independent procedures were correlated (Spearman’s rho = 0.50, *P* < 0.01); this conclusion was also supported by the significant association between the amount of OC and INM mucus obtained from each subject (Spearman’s rho = 0.56, *P* < 0.01, Supplementary Fig. 1a), suggesting that the individual variability in the amount of collected mucus was caused by an intrinsic factor(s).

Individual variation in the INM mucus amounts was associated with intrinsic factor(s) and the environmental factor of humidity on the day of sampling. The amount of INM mucus appeared to be lower when collected in sunny weather (relative humidity, 42%) than that in rainy weather (71–100%), suggesting dehydration of the INM mucus (Supplementary Fig. 1b). In contrast, the amount of OC mucus collected did not differ according to weather. The weather dependency of INM mucus was significant when analyzed as normalized values with individually varied amounts of OC mucus (Fig. 1c). Thus, INM mucus is important in humidifying the inhaled drier air before it reaches and dehydrates the OC mucus; otherwise, the functions of the OC mucus will be impaired, as reported in the tracheobronchial mucosa^38^. Meanwhile, weather independence suggests that individual variation in the amount of OC mucus is mainly attributable to intrinsic factor(s).

### Solute concentrations in OC mucus

There has been little description of the basic properties of OC mucus, especially the concentrations of total protein and inorganic elements. In this study, we first determined the total protein concentration of the sampled bodily fluids using a Pierce^TM^ bicinchoninic acid (BCA) assay (Fig. 1d). The OC mucus contained higher protein concentrations than either INM mucus or saliva. Notably, the protein concentration of OC mucus also varied considerably between individuals, ranging from 6.2 to 20.8 mg/mL, and showed a negative relationship with the collected amount of OC mucus (Fig. 1e).

High xenobiotic metabolism requires a high concentration of enzymes and inorganic elements that constitute their active centers. Concentrations of four trace bioinorganic elements (Mg, Fe, Zn, and Cu) were successfully quantified from all samples, except for Cu from the saliva. The concentrations of five other elements, Al, Cr, Mn, Ni, and Co, were below the lower limit of quantification (100 ppb for the OC and INM mucus and 5 ppb for the saliva). Overall, the OC mucus contained higher concentrations of inorganic elements than did either the INM mucus or saliva, except for Zn (Fig. 1f–i). The concentration of Zn did not differ between the OC and INM mucus and showed female-specific enrichment (Fig. 1j). In agreement with the purpose of xenobiotic metabolism and detoxification, we detected individually varied concentrations of a low-molecular-weight antioxidant, glutathione^39^, in the OC mucus (Fig. 1k). The concentration of glutathione was correlated with Fe concentration (Supplementary Fig. 1c). Similar concentrations of glutathione were detected in a mixture of equal amounts of the INM mucus from participants: 163 μM of oxidized glutathione (GSSG) and 23 μM of reduced glutathione (rGSH). In contrast, the saliva showed no detectable levels of glutathione.

The high concentration of Cu in the OC mucus was remarkable even when compared with that in the serum (mean concentration: 4.8 ppm (75 μM) in OC mucus vs. 1.1 ppm in serum), but similar to that in mouse OC mucus estimated by measuring nasal lavage fluid (42 μM)^15,40^. This result appears to be reasonable given the fact that OSNs utilize Cu for the sensitive detection of sulfur odorants^15^. The Cu concentration in the OC mucus ranged from 12.6 to 189 μM, similar to the concentration range effective for enhancing the responsiveness of OR-expressing cells to a sulfur compound, *tert*-butyl mercaptan (*t*BM; Fig. 1l)^41^. However, this variable did not explain the individual variance in olfactory sensitivity to *t*BM (Fig. 1m). This discrepancy suggests: (1) a methodological limitation for discriminating protein-bound Cu ions and free Cu ions; and (2) the presence of more dominant factor(s), such as genetic variation of ORs detecting sulfur odorants, as suggested previously^42^.

### Odorant-capturing property of olfactory mucus

An important functional aspect of the OC mucus has been proposed based on the functions of OBPs. It has been considered that the purpose of their odorant-binding ability is to efficiently solubilize hydrophobic odorants and deliver them to ORs^9,22,43,44^. However, this is based only on in vitro analyses using artificially produced OBPs; therefore, it remains unknown whether the OC mucus itself has this ability and contributes to olfactory perception of bound odorants.

**Figure 2:**
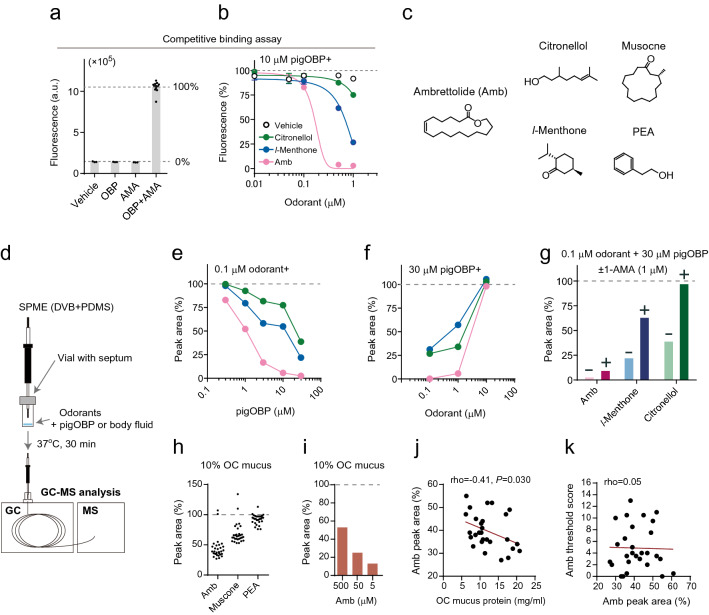
Odorant-capturing property of the olfactory mucus. (**a**) Competitive binding assay for pigOBP to odorants. Data represent fluorescence from vehicle (Tris–HCl), 10 μM pigOBP, 1 μM 1-AMA, and their mixture. (**b**) Change in fluorescence when pigOBP-1-AMA complex was exposed to increasing concentrations of odorants. Fluorescence was normalized using values from the vehicle and mixture of pigOBP and 1-AMA. Error bars, standard deviation over three replicates. (**c**) Chemical structures of odorants. (**d**) Experimental procedure for the quantification of odorant-binding capacity based on headspace concentrations of odorants. (**e**, **f**) Odorant-capturing capacity of pigOBP. Three odorants emitted from pigOBP solution were extracted and analyzed with SPME-GC/MS. (**e**) pigOBP reduced the release of odorants from a solution in a dose- and affinity-dependent manner. (**f**) Odorant-capturing properties of 30 μM pigOBP depending on odorant concentration. (**g**) Addition of 1-AMA-induced release of odorants from pigOBP solution. Data are shown as percent peak area, where the detected peak area from odorant solution without pigOBP was set to 100%. (**h**) Individual variance of the odorant-capturing capacity of OC mucus. Relative headspace concentration of odorants emitted from × 10 diluted OC mucus. Odorants were added with ethanol for a final concentration of 500 µM. The peak area of each odorant without OC mucus was set to 100% after normalization with the peak area of ethanol. (**i**) Odorant-concentration dependence of the capacity of the OC mucus. Relative headspace concentration of odorants emitted from × 10 diluted OC mucus (5–500 μM for final concentrations). (**j**) Correlation of odorant-capturing capacity and protein concentration of the OC mucus (n = 28). (**k**) Association between odorant-capturing capacity and perceptual sensitivity to Amb. OBP, odorant binding protein; PEA, phenylethylalcohol.

At least two putative OBPs were expressed in human olfactory mucus, but their odorant-binding activity was not characterized. In this study, we evaluated the odorant-capturing activity of OC mucus. Prior to investigating the OC mucus, we used *Sus scrofa* odorant-binding protein-1 (pigOBP), a well-characterized mammalian OBP, to establish the experiment^45,46^. First, purified recombinant pigOBP was subjected to a well-established competitive binding assay using the fluorescence ligand 1-aminoanthracene (1-AMA)^44–46^. The result of a previous study in which fluorescence from pigOBP and 1-AMA complex was quenched upon binding of a known ligand, citronellol, was confirmed (Fig. 2a–c)^46^. Using this binding assay, ambrettolide (Amb) and *l*-menthone were identified as novel ligands with a higher affinity for pigOBP (Fig. 2b, c). As the competitive binding assay requires a large amount of OC mucus to test multiple odorants, a different type of experiment was employed. The pigOBP solution was prepared in a glass vial and mixed with three ligands (Fig. 2d). The amount of ligands released from the pigOBP solution into the headspace was absorbed and measured using solid-phase microextraction (SPME) followed by gas chromatography-mass spectrometry (GC/MS). Ligands with a higher affinity to pigOBP in the competitive binding assay showed slower release, resulting in lower concentrations in the headspace (Fig. 2e, f). The slower release was caused by the odorant-capturing property of pigOBP, as it was blocked by the addition of the competitive ligand 1-AMA (Fig. 2g). These data validated the experiment for evaluating the odorant-capturing activity of mucus samples based on the amount of odorant in the headspace.

The odorant-capturing properties of a mixture of equal volumes of OC mucus obtained from 30 participants were investigated. The following three odorants were tested: (1) Amb, which has the highest affinity for pigOBP; (2) muscone, an important fragrance due to its superior odor quality; and (3) phenylethylalcohol (PEA), the most common odorant used for olfactory research due to its small trigeminal effect^47^. The headspace concentrations of the three odorants were markedly lower when mixed with the OC mucus than with INM mucus, saliva, or saline, although we were unable to produce assay replicates required for statistical analysis due to the limited amount of collected samples (Supplementary Fig. 2). The capacity was still clear for Amb and showed individual variability even when the OC mucus was applied at ten-fold dilution (Fig. 2h). This slowed release was dependent on the concentration of Amb mixed with OC mucus, consistent with the observed characteristics of pigOBP (Fig. 2i). We can exclude the possibility that the lower concentration of Amb in the headspace was caused by decomposition in the OC mucus. This is because Amb remaining in the OC mucus was detectable when extracted with an organic solvent and analyzed by GC/MS (81% of Amb extracted from saline solution). The Amb-capturing property was positively correlated with the protein concentration in the OC mucus (Fig. 2j).

We then investigated whether the odorant-capturing activity of OC mucus is associated with perception, and the results showed that it was not a determinant for the sensitive detection of Amb because no correlation was observed between them (Fig. 2k). The two participants whose OC mucus completely lacked the capturing activity for Amb showed an average perceptual sensitivity score compared with 30 participants (7.5 and 3, within 0–15; average score: 4.9). In summary, this study provides evidence that human OC mucus exhibits significant odorant-binding capacity not associated with olfactory sensitivity.

### Odorant conversions by enzymes in the olfactory mucus

**Figure 3:**
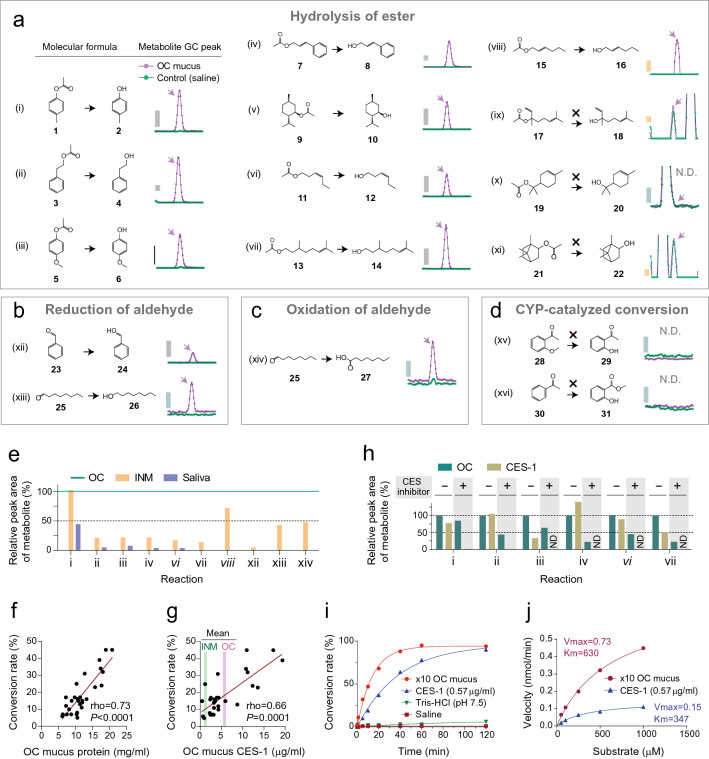
Enzymatic activity of the olfactory mucus. (**a–d**) Assumed enzymatic activity of olfactory cleft (OC) mucus and the detection of resulting metabolites. Odorants were incubated in saline or OC mucus. The reactant was extracted using ethyl acetate and analyzed using gas chromatography/mass spectrometry (GC/MS). Intensity of total ion or extracted ion peaks was normalized to a peak area of ester **19**, which did not undergo OC mucus-mediated conversion. Grey and blue bars represent normalized total ion intensities of 20,000 and 2000, respectively. Yellow bar represents an extracted ion intensity of 50. (**e**) Comparison of enzymatic activity of the OC mucus with inferior nasal meatus (INM) mucus and saliva. The amounts of product in each enzymatic conversion are presented as normalized values, where a peak area of metabolite in OC mucus is set to 100%. (**f**) Individual difference in enzymatic activity of OC mucus and its association with the concentration of protein. Esterase activity of individual × 10 OC mucus was evaluated based on a conversion rate as [*p*-cresol (mol)]/([*p*CA (mol)] + [*p*-cresol (mol)]) 5 min after *p*-cresyl acetate (*p*CA) was applied to OC mucus (n = 29). (**g**) Association between esterase activity and carboxylesterase 1 (CES-1) concentration in OC mucus (n = 29). Green and magenta lines denote mean values of INM and OC mucus, respectively. (**h**) Substrate selectivity of OC mucus and CES-1. Recombinant CES-1 did not show identical reactivity with OC mucus. OC mucus showed residual activity under CES inhibitors (bis(4-nitrophenyl) phosphate), which completely inhibited CES-1. Inter-sample variability of the detected peak area was corrected using the peak area of non-metabolized ester **19**. (**i**) Time course of the conversion rate from *p*CA to *p*-cresol in × 10 diluted OC mucus or CES-1 solution. The reaction proceeded linearly up to 5 min after the addition of *p*CA in the ten-fold diluted OC mucus and up to 40 min in the CES-1 solution. (**j**) Michaelis–Menten plot of *p*CA metabolism in × 10 diluted OC mucus or CES-1 solution. Various concentrations of *p*CA were added, and the conversion rate was measured at each time. ND, not detected.

Next, we describe the metabolic capacity of human OC mucus and its individual variations. First, six odorants were selected for our experiments because they were reported to be metabolized in OC mucus-related samples (esters: *p*-cresyl acetate [*p*CA] **1** and *trans*-2-hexenyl acetate **15**; aldehydes: benzaldehyde **23** and octanal **25**; ketones: 2′-methoxyacetophenone **28** and acetophenone **30**)^7,20,48,49^. These odorants were mixed with the collected OC mucus (a mixture of equal amounts from all participants), and their ethyl acetate extracts were analyzed using GC/MS. The results showed that the OC mucus induced the following metabolisms: hydrolysis of the esters producing *p*-cresol **2** (99% as total ion chromatogram (TIC) peak abundance ratio of metabolite) and *trans*-2-hexenol **16** (16%); and reduction and oxidation of the aldehydes producing benzylalcohol **24** (51%), octanol **26** (33%), and octanoic acid **27** (50%; Fig. 3a–d, Supplementary Fig. 3a). However, metabolites derived from the two ketones, 2′-hydroxyacetophenone **29** and methyl salicylate **31**, were not detected, even though the gene expression of CYPs, which oxidize them, has been reported in the OC^50^.

The remarkable capacity of OC mucus for the hydrolysis of esters led us to investigate the structure–activity relationship. An additional experiment was conducted to test a series of ester odorants, with *p*CA as a positive control (97% as TIC peak abundance ratio of metabolite). The results demonstrated that the OC mucus converted phenolic esters (2-phenylethyl acetate **3** [43%], anisyl acetate **5** [8%], cinnamyl acetate **7** [16%]), a cycloaliphatic ester (*l*-menthyl acetate **9** [14%]), and aliphatic esters (*cis*-3-hexenyl acetate **11** [19%], citronellyl acetate **13** [25%]; Fig. 3a). In contrast, sterically hindered esters (linalyl acetate **17**, terpinyl acetate **19**, and isobornyl acetate **21)** were not converted. The observed metabolism was induced more prominently by the OC mucus than by either INM mucus or saliva, consistent with the higher solute concentrations in the OC mucus (Fig. 3e, Supplementary Fig. 3b).

Next, the individual variability in the esterase activity of OC mucus was analyzed by focusing on the conversion of *p*CA to *p*-cresol. The comparison was conducted based on a conversion rate of 5 min, as the reaction proceeded linearly for up to 5 min after the addition of *p*CA (see Fig. 3i). One participant was excluded from this analysis because the amount of mucus collected was insufficient. The conversion rates (molar ratio) differed considerably between participants and ranged from 5 to 45%, with a mean value of 18%. The esterase activity was negatively correlated with the amount of OC mucus and positively correlated with the concentrations of various components, such as total protein (Fig. 3f, Supplementary Table 1).

The molecules causing high esterase activity in the OC mucus were also investigated. CES-1 was presumed to contribute to this reaction in a previous study using rodents^7^. An enzyme-linked immunosorbent assay (ELISA) showed that the CES-1 concentration was higher in the OC mucus than in either the INM mucus or saliva (mean concentration: 5.73 μg/mL, 1.24 μg/mL, and 0.06 μg/mL, respectively, Fig. 3g). However, CES-1 did not fully account for esterase activity in the OC mucus. CES-1 concentration in the OC mucus accounted for less individual variance in esterase activity than the concentration of total proteins (44% vs. 53%, Fig. 3f, g). In addition, the OC mucus showed different substrate selectivities than the recombinant CES-1 solution. Recombinant CES-1 did not reconstitute the reactivity of the OC mucus to anisyl acetate **5** and citronellyl acetate **13** (Fig. 3h). Moreover, the OC mucus showed residual activity under the CES inhibitors benzil and bis(4-nitrophenyl) phosphate (BNPP), which completely inhibited reactions mediated by an equal concentration of recombinant CES-1 (Fig. 3h, Supplementary Fig. 3c). Finally, a solution with an equivalent concentration of recombinant CES-1 showed less reactivity than OC mucus on enzyme kinetics analysis (Fig. 3i, j). These results indicated the presence of another enzyme(s) responsible for enzymatic activity.

### Perception of odorant metabolites

**Figure 4:**
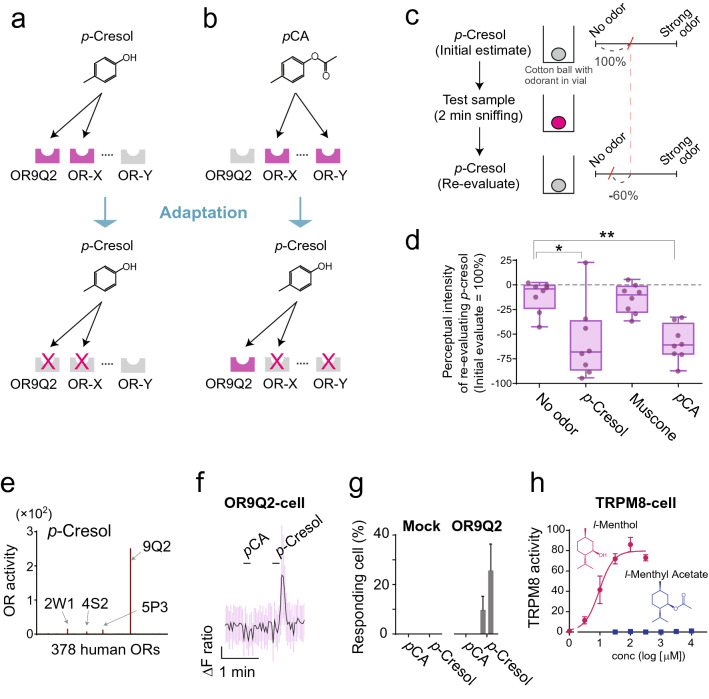
Effect of the enzymatic conversion on perception. (**a**, **b**) A model showing olfactory adaptation for *p*-cresol or *p*-cresyl acetate (*p*CA) through activation and desensitization of a distinct subset of olfactory receptors (ORs). (**c**) The experimental adaptation scheme and sensory evaluation. Each subject evaluated the perceived intensity of the first bottle and the third bottle, both of which contain cotton balls saturated with *p*-cresol. The second bottle contained an adapting stimulus: distilled water (no odor), *p*-cresol, muscone, or *p*CA. Participants were asked to smell *p*-cresol and record their perceived intensity of the odor on a 95-mm scale, marked from no odor to strong odor. (**d**) Change in perceived intensity of *p*-cresol after prolonged exposure with each test sample (n = 8). Statistically significant changes in comparison with a control (no odor) were determined by one-way analysis of variance (ANOVA) with unequal standard deviation and Holm-Sidak’s multiple comparisons test. *, *P* = 0.0303; **, *P* = 0.0012. (**e**) Screening of human ORs for *p*-cresol. Each of the 378 ORs listed along the x-axis was expressed in HEK293T cells and stimulated with *p*-cresol (1 mM). OR activations were monitored using a CRE-regulated luciferase reporter gene assay. The y-axis indicates the fold increase of OR activity where a signal from stimulated cells is divided by that from non-stimulated cells expressing the same OR (mean values from two screening replicates). (**f**, **g**) Calcium imaging of OR9Q2 activation. (**f**) A representative response trace of responsive cells. The purple vertical lines around the trace represent the standard deviation from 50 cells. Horizonal lines above the trace indicate the duration of stimulations with 1 mM *p*CA and 0.3 mM *p*-cresol. (**g**) Proportion of responding cells to 0.3 mM or 1.0 mM of each odorant from three independent experiments (mean ± SE). (**h**) Dose–response curve of transient receptor potential melastatin 8 (TRPM8) activity with *l*-menthol and *l*-menthyl acetate. Calcium ion influx-dependent fluorescence in TRPM8 stably expressed in HEK293T cells was measured. The fluorescence intensity was normalized with 4 μM ionomycin. Data are from three independent experiments (mean ± SE).

A previous study reported that a low rate of enzymatic conversion of an odorant in human saliva slightly affected odor perception^8^. We hypothesized that the drastic esterase activity of the OC mucus had a larger effect on the odor perception of the substrate. We conducted a sensory study using an olfactory adaptation paradigm based on the previous study^8^. Olfactory adaptation is a widely known phenomenon of odorant-specific reduction in sensitivity following prolonged exposure to an identical odorant (Fig. 4a, b)^51^. Accordingly, in the current study, olfactory sensitivity to *p*-cresol decreased after pre-exposure to *p*-cresol, whereas it was not induced by prolonged smelling of muscone (Fig. 4c, d). In contrast, pre-exposure of *p*CA induced a significant reduction in the perceptual intensity of its metabolite *p*-cresol, suggesting that *p*CA is converted to *p*-cresol in OC mucus. An alternative explanation for this result is that smelling *p*CA induced desensitization of the OR, which plays a major role in the recognition of *p*-cresol. However, the results of our in vitro assays ruled out this possibility. Among the 378 tested human ORs expressed in HEK293T cells, OR9Q2 was found to be the most sensitive receptor for *p*-cresol (Fig. 4e, Supplementary Fig. 4). Subsequent functional characterization showed that OR9Q2 was not sensitive to *p*CA (Fig. 4f, g). Thus, the reason why adaptation with *p*CA parallels an exceptional adaptation with *p*-cresol is that the conversion of *p*CA into *p*-cresol occurs in the nasal cavity. However, it is still unclear whether humans perceive a mixture of metabolizing *p*CA and generating *p*-cresol within a sniff of *p*CA, owing to a lack of information about reaction kinetics in vivo.

A sniff in humans lasts 1–2 s. We examined whether the reaction kinetics of enzymatic conversion was sufficiently rapid to influence perception within a sniff. Given that it is difficult to evaluate the generation of a specific odor quality derived only from a product mixed with a substrate, a familiar somatosensory perception, that is, cooling, was used as a clearer index. Sniffing *l*-menthol **10** or its acetate, *l*-menthyl acetate **9,** elicits a cooling sensation, which is clearly evaluable^52^. The cooling sensation is most likely caused by the activation of transient receptor potential melastatin 8 (TRPM8) in somatosensory neurons^53^. In contrast to *l*-menthol **10**, *l*-menthyl acetate **9** showed no TRPM8 activity (Fig. 4h). This discrepancy presents evidence for the presence of OC mucus activity in vivo that metabolizes *l*-menthyl acetate **9** to produce *l*-menthol **10** as demonstrated using sampled OC mucus (Fig. 3a). More importantly, this result demonstrates that the enzymatic conversion reaction is sufficiently quick to affect odor perception within a sniff. Thus, our perceptual snapshot of the chemical nature outside the nose is editted by the olfactory mucus before we perceive it.

### Relationship between decline in olfaction and aging-related changes in the OC mucus

**Figure 5:**
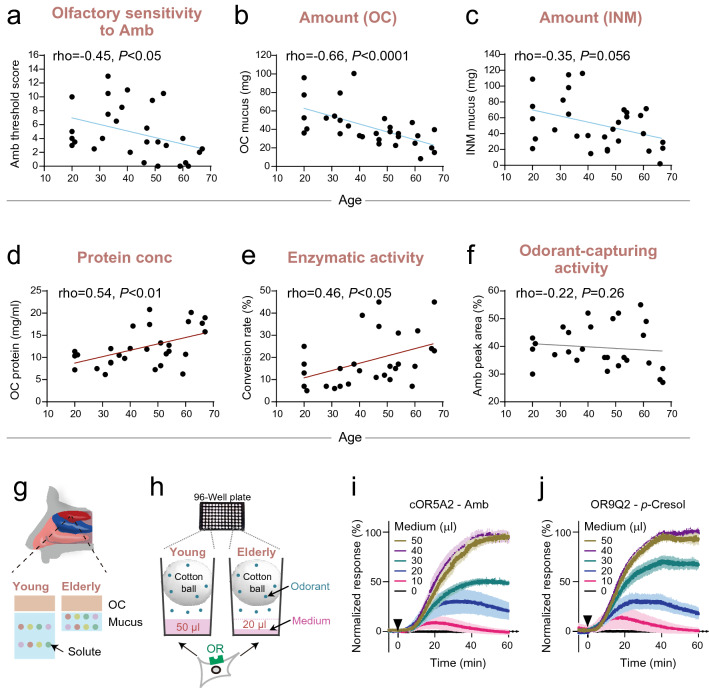
Relationship between the decline in olfaction and change in olfactory cleft mucus with aging. (**a–f**) Factors associated with age. The x-axis indicates the age of participants, and the y-axis represents olfactory threshold values for ambrettolide (Amb; **a**, n = 28), amount of olfactory cleft (OC) mucus (**b**, n = 30), amount of inferior nasal meatus (INM) mucus (**c**, n = 30), protein concentration in the OC mucus (**d**, n = 30), conversion rate from *p*-cresyl acetate (*p*CA) to *p*-cresol in × 10 diluted OC mucus (**e**, n = 29), and relative headspace concentration of Amb emitted from the OC mucus (**f**, n = 28). (**g**) Graphical summary of age-dependent changes in olfactory mucus. The OC mucus decreased in amount and increased in the concentration of components with aging. (**h**) Experimental procedure for assaying the activation of olfactory receptor (OR)-expressing cells covered with different amounts of medium (0–50 μL), which mimic the age-dependent decrease in the amount of OC mucus. Cotton balls saturated with odorants (100 μM) were placed above cells in each plate well. (**i**, **j)** Response of cOR5A2 or OR9Q2-expressing cells against Amb or *p*-cresol diffusing from the vapor phase. The response was normalized with the maximum response in the 50 μL condition. Each line shows the average response from three independent experiments. The vertical shades represent standard errors.

Olfactory sensitivity generally decreases with age. As we tested younger participants (age range: 20–67 years) than those in previous studies that showed a significant age-related decline^24,26^, a negative relationship between age and olfactory sensitivity to two odorants (PEA and *t*BM) was not detected (Supplementary Fig. 5). In contrast, the current study found an age-dependent reduction of perceptual sensitivity to Amb (rho = − 0.45, *P* < 0.05, Fig. 5a). This result indicated that our OC mucus samples were collected from a group of participants with age-related decline in olfaction. Therefore, it was possible to identify a candidate factor from the OC mucus samples that showed age-dependent changes and reduced olfactory sensitivity.

Various age-related factors have been proposed to potentially cause a decline in olfactory sensitivity^10,27–31,33–35,37^. However, there are still no examples of mechanical explanations linking changes in these candidate factors to decreased olfactory sensitivity. This study found novel age-dependent changes. The aforementioned individual variability in the OC mucus amount was significantly correlated with age (rho = 0.66, *P* < 0.001, Fig. 5b). Elderly participants showed a 60% lower amount of OC mucus than younger participants (average: 59.1 ± 21.0 mg in participants aged in their 20 s vs. 23.5 ± 10.6 mg in participants aged in their 60 s). The INM mucus also decreased in an age-dependent manner (rho = − 0.35, *P* = 0.056) and, therefore, no longer functioned sufficiently to humidify the OC mucus (Fig. 5c). In contrast, the protein concentration and enzymatic activity increased, indicating that the reduced amounts of OC mucus were caused by a decrease in water content (Fig. 5d, e). Despite the aging-related increase in concentrations of total proteins in the OC mucus, the capturing activity for Amb was not enhanced (Fig. 5f), likely due to a specific decrease in putative OBPs and/or the existence of other unknown factor(s) associated with the activity^10^.

The increase in solute concentrations and enzymatic activity with aging may explain the impairment of odor identification skills against a restricted range of odorants such as sulfur and esters; however, it does not explain the impairment of olfactory sensitivity against Amb (Fig. 5a) and other general odorants. Instead, a decrease in the amount of the OC mucus more likely accounts for the decline in olfactory sensitivity to general odorants owing to dehydration-mediated damage to the lipid bilayer of OSNs and distortion of OR structures. This is consistent with previous studies. One study demonstrated that a reduced amount of olfactory mucus in mice decreased olfactory sensitivity^54^. Another study reported that increasing the mucus amount of newborn rabbits resulted in elevated olfactory sensitivity^16^.

The current study reconstituted the effect of a decrease in the amount of OC mucus on OR activation in response to odorants (Fig. 5g, h). In contrast to a large number of previous studies in which odorants were stimulated in the liquid phase^8,15,19,41^, HEK293T cells expressing each OR were presented with an odorant in the vapor phase, allowing us to evaluate OR activation under more practical conditions. A cotton ball saturated with an odorant solution was placed above the cells in each well of a 96-well plate (Fig. 5h). Odorants volatilized from the cotton ball reached the OR-expressing cells through the medium, and their responses were monitored using real-time GloSensor™. Vapor-phase stimulation was observed as indicated by the finding that no response was observed when a non-volatile cellular stimulant (i.e., forskolin) was tested. Activations of a consensus version of OR5A2 (hereafter, cOR5A2) and OR9Q2 against the vapor phase of Amb and *p*-cresol were monitored^55^. The result showed that a low volume of medium covering OR-expressing cells caused a lower maximum response; a 60% reduction in the amount of medium (from 50 to 20 μL) decreased the response amplitude of cOR5A2 and OR9Q2 to 33% and 35%, respectively (Fig. 5i, j). This experiment did not reconstitute the physical and biochemical properties of OC mucus. Considerable toxicity of OC mucus to HEK293T cells hampered the experiment in more physiological conditions. Therefore, we cannot exclude the possibility that differences in OC mucus amounts resulted in differential effects on OR activation. Nonetheless, this is the first study to propose a causative factor for the age-dependent decline in olfactory sensitivity with a potential mechanistic explanation.

## Discussion

The present study extensively described the characteristics of human OC mucus. OC mucus contains higher concentrations of solutes, including total proteins, inorganic elements, and glutathione, than other bodily fluids. Our functional analyses provide conclusions regarding the previously suggested functions of OC mucus and its contribution to scent perception. OC mucus exhibits a remarkable odorant-capturing capacity, but this does not explain perceptual sensitivity to an odorant. In addition, the current study found that odorant metabolism in OC mucus is involved in perception. Finally, the present results demonstrate an age-dependent decrease in the amount of OC mucus as a potential cause of age-related decline in olfactory sensitivity.

This study revealed the concentrations of solutes in OC mucus and their individual variability. Our results provide essential information that accounts for previous implications of OC mucus functions, such as odorant-binding capacity, high xenobiotic metabolism, and Cu-mediated odor recognition^9,41,43^. Moreover, the present data, together with the previously reported protein composition, enable the reconstitution of OC mucus and help investigate the functional significance of the emerging properties, including Zn as a d-block element and as a sex-specific characteristic^56,57^. The assumed functions of OC mucus are not limited to olfaction and include the protection of OSNs from harmful compounds and the prevention of infection. The disclosed raw data will provide a basis for future studies to understand the functions of olfactory mucus from multiple perspectives (Supplementary Table 1).

The most widely assumed function of OC mucus is the transportation and concentration of odorants via OBPs. Indeed, a recent study tested the potential function of a human OBP in odorant recognition by ORs; however, they tested an artificially produced OBP without any ligands. Therefore, the question of whether OC mucus has the capacity to capture odorants and promote their sensing remained unanswered. Our in vitro and in vivo experiments concluded that OC mucus had a drastic odorant-binding capacity; however, they did not explain individual variance in olfactory sensitivity. This is consistent with the results of an insect model that can respond to odorants with comparable sensitivity when all OBP genes are deleted^58^. We speculate that the odorant-capturing activity of OC mucus may contribute to the rapid removal of odorants from the microenvironment of OSNs to ensure recovery from prolonged adaptation and prevent damage caused by the accumulation of harmful substances^59^.

This study revealed various high xenobiotic metabolisms of OC mucus with kinetic analysis. Two recent studies have reported the enzymatic activities of human OC mucus-derived samples against select odorants^8,14^; however, these studies evaluated the samples under artificial conditions. One study obtained OC mucus as a saline solution from an irrigated nasal cavity, which likely caused dilution and inclusion of impurities from surrounding regions, such as mucus from the inferior nasal meatus (INM)^8^. Indeed, this study did not detect a relatively higher enzymatic activity of OC mucus. Another study directly collected OC mucus but aldehyde reduction was only detected in diluted OC mucus supplemented with co-enzymes^14^. The current study tested OC mucus without dilution or any treatments and investigated its activity against a wider range of odorants. Notably, individual differences in enzymatic activity were determined based on reaction kinetics, providing more precise information than a previously reported comparison based on endpoint measurements^8,14^. Our results demonstrated that intact OC mucus has an outstanding capacity for odorant conversion. We note that the lower capacity of INM mucus per unit mass than OC mucus likely plays significant contribution to perception given the fact that INM mucus provides wider surface area for enzymatic conversion of inhaled odorants. This capacity clarification may allow for the design of a pro-odorant, which activates an OR only after achieving a sufficient level of enzymatic conversion.

The most important finding in this study was an age-dependent decrease in the amount of OC mucus. Consistent with our observations, previous studies have shown that aging induces abnormalities in water homeostasis and results in a thinner mucus, with deteriorated function in other tissues^60–62^. Age-related degeneration of the Bowman’s glands and mucus secretion was observed in the nasal cavity^28^. The decrease in water content and increase in solute concentrations probably not only causes dryness-mediated dysfunction of OSNs, but also leads to an increase in the viscosity of the mucus layer. This lowers the diffusion velocity of absorbed odorants and consequently worsens the detection efficiency of odorant molecules by OSNs. An increase in mucus concentration (i.e., dehydration) is known to decrease the speed of mucus flow and remove substances, including odorants^63,64^. The slower removal and decreased volume of mucus may synergistically increase the accumulation of harmful volatiles and infectious microorganisms, which impairs the neuroepithelium and Bowman’s glands, resulting in the acceleration of senescence. The present evidence suggests that the recovery of water in OC mucus may be an effective treatment for olfactory dysfunction, a cause of impaired QOL in the elderly.

## Materials and methods

### Human participants

The participants were recruited by snowball sampling and were compensated. Thirty Japanese adults aged between 20 and 60 years participated in the study. The participants did not have any subjective or objective evidence of sinonasal inflammation based on their history or nasal endoscopy. Pregnant women were excluded. The study protocol was approved by the ethical review boards of Kao Corporation and Edogawa Hospital (approval number: T141-180620) and was conducted according to the Declaration of Helsinki principles. All participants provided informed consent.

### Odor threshold tests

Olfactory thresholds for three compounds (PEA, Amb, and *t*BM) were measured. Purchase sources of the odorants are shown in Supplementary Table 2. Sensory thresholds were collected using odorants diluted with odorless mineral oil (Sigma-Aldrich, St. Louis, MO, USA) using a three-alternative forced-choice procedure. The highest concentrations of odorant solutions were as follows: PEA at 1000 ppm; Amb at 10,000 ppm; and *t*BM at 1 ppm. They were diluted two-fold 15 times to prepare 16 dilutions of each odorant. In each trial, participants were presented with three sets of bottles in random order: one set contained the odorant stimulus, and the others only contained the diluent (blanks). After sniffing each set sequentially, the participants were asked to identify the odor-containing bottle. No recognition or quality identification was required. For every incorrect detection, the next-lowest concentration was presented. The first trial started with the lowest concentration, and for two consecutive correct detections, a four-times higher concentration was presented in the second trial. For two consecutive correct detections in the second trial, the next-highest concentration was given until the odorant was correctly identified. When the first concentration of odorant was not identified in the second trial, the next-lowest concentration was presented. The average concentrations of the first and final correct detections were identified as the threshold score. The concentrations were presented as the dilution times of the solutions (15–0). The detection of the lowest concentration was scored as 15, and the misdetection of the highest concentration was scored as 0. There was a mistake in the protocol during the threshold tests for two participants, and their threshold scores were excluded.

### Collection of saliva and mucus

Whole saliva was collected directly into a plastic tube after rinsing the mouth with water. The sample was centrifuged (10,000 rpm for 10 min) and frozen at – 80 ℃ until needed. Whole saliva was collected on the same day as the odor threshold test, and olfactory mucus was collected 1–3 weeks later at the Edogawa Hospital. Mucus samples were collected using neurosurgical pads (BEMSHEETS XR, 0.7 × 0.7 cm; KAWAMOTO Corporation, Osaka, Japan). The pad could absorb a maximum of approximately 150 mg of water, and the amount of all the collected mucus was below 150 mg. The pads were then placed in a tube with a hole in the bottom made by a 20-gauge needle. Another tube was placed below the punctured tube and centrifuged (10,000 rpm for 10 min). The collected nasal secretions were frozen at − 80 ℃ until use. In one subject, no INM mucus was collected from either the left or right nasal cavities. In one subject, the pad placed on the left INM dropped down to the mouth during collection. Only the INM mucus collected from the right nasal cavity was used for all analyses. Olfactory mucus was collected for 5 days (6 participants/day). During this time, the weather on one day was sunny and on the other days was rainy.

### Protein, human carboxylesterase 1 (CES-1), and glutathione concentrations

Protein concentrations were determined using the BCA protein assay with BSA as a standard. The concentrations of CES-1 were determined using a CES-1 ELISA kit (RayBiotech; Peachtree Corners, GA, USA). Glutathione concentrations were determined using a GSSG/GSH quantification kit (DOJINDO, Kumamoto, Japan). Equal amounts of mucus collected from the left and right sides were mixed and analyzed except for one sample of INM mucus, as previously described.

### Determination of inorganic element concentration by inductively coupled plasma mass spectrometry

*N*-methyl-2-pyrrolidone (NMP; Kishida Chemical Co., Ltd., Osaka, Japan; for the electronics industry) and nitric acid 1.38 (60%; Kanto Chemical Co., Inc., Tokyo, Japan; for the electronics industry) were used to prepare the calibration curve solution and sample dilution. SPEX CertiPrep (XSTC-13 elemental mixed standard solution, 10 μg/mL, 5% HNO_3_; SPEX, Metuchen, NJ, USA) was used as the standard solution for the calibration curve. A mixture of 5 μL of OC mucus and INM mucus (left and right mix) or 100 μL of saliva supernatant was dissolved in 5 mL 1% nitric acid-containing NMP. Automatic measurements were performed using a Thermo Fisher Scientific (Waltham, MA, USA) inductively coupled plasma mass spectrometer (ICP-MS) iCAP Qs connected to the automatic measurement sampler ESI SC2 DX and controlled by the software Qtegra. A sample diluted with 1% nitric acid-containing NMP was directly introduced into a quartz cyclone chamber set at 4 ℃ by self-aspiration. Oxygen was introduced into the plasma to prevent the accumulation of carbon. Helium gas collision conditions were applied for Zn measurement. Cold plasma and 1% NH_3_-He gas collision reaction conditions were applied to all elements except Zn (Supplementary Table 3).

The calibration curve was prepared with a 1% nitric acid-added NMP solution at concentrations of 0, 0.1, 0.2, 0.5, 1, 5, 10, 50, and 100 ng/mL. Linearity (r > 0.999) was obtained for all elements, and the lower limit of quantification of the injected sample was confirmed to be 0.1 ng/g (100 ppb for the OC and INM mucus, 5 ppb for saliva). The samples were too concentrated to determine Na, K, and Ca concentrations; thus, standard curves were prepared in the range of 0.1 ppb to 100 ppb for Mg and 0.1–10 ppb for the others. Blank samples were prepared by soaking neurosurgical pads in Milli-Q water and then collecting the water as olfactory mucus. Concentrations of Mg, Fe, Cu, and Zn in the mucus or saliva samples were higher than those in the blank samples. The Fe concentration of one OC mucus sample was higher than that of the others (~ 40 ppm, while the average of the others was 1.67 ppm). This sample might have contained a small amount of blood; thus, the ICP-MS data of this sample were excluded from the analyses.

### Expression vector for pigOBP

DNA coding for pigOBP was synthesized using GenScript gene synthesis services (GenScript Biotech Corp., Piscataway, NJ, USA). The synthesized gene is basically the same sequence as NM_213796.1; however, it encodes proteins bearing the F88W mutation^46^ and His6 tag at the C-terminus but without the 15 amino acid sequence that encodes a signal peptide sequence at the N-terminal. The synthesized gene was digested with *Eco*RI and *Xho*I restriction enzymes and cloned into pET-22b (Merck Biosciences, Madison, WI, USA). This resulted in the final expression vector encoding pigOBP with an N-terminal pelB leader sequence. pigOBP was expressed and purified by GenScript using *Escherichia coli* strain BL21(DE3) and a Ni column. The concentration was determined using the Bradford protein assay with BSA as the standard. pigOBP was detected using mouse-anti-His mAb (GenScript) in western blot analysis.

### Fluorescence binding assay

The measurements were performed as previously described^46^. Odorant solutions (Amb, *l*-menthone, and citronellol) were prepared as 100-mM stock solutions in EtOH. 1-AMA (Accu Standard Inc., New Haven, CT, USA) was prepared as a 1 mM stock solution in EtOH. pigOBP was prepared as a 60 μM stock solution in phosphate-buffered saline. Stock solutions were frozen and then diluted with 50 mM Tris–HCl (pH 7.5) for the assays. We added 50 µL of the tested solution to a 96-well black plate (Corning Inc., Corning, NY, USA). The fluorescence emission of the solutions was recorded on an EnSight Multimode Plate Reader (PerkinElmer, Waltham, MA, USA).

### Odorant-capturing property of pigOBP determined by headspace gas chromatography-mass spectrometry

The odorant mixture solution (40 μL) containing Amb, *l*-menthone, and citronellol was prepared in 50-mM Tris–HCl (pH 7.5) with or without various concentrations of pigOBP. The solution was then pipetted into a 2-mL headspace vial (Agilent Technologies, Santa Clara, CA, USA), sealed with a polytetrafluoroethylene (PTFE)/silicone septum screw-cap, and heated for 5 min at 37 °C in a water bath to expose the SPME fiber (polydimethylsiloxane [PDMS]/divinylbenzene [DVB], df 65 μm, SUPELCO, Inc., Bellefonte, PA, USA) to the headspace of the vial for 30 min. The SPME devices were conditioned before sampling. An Agilent 7890A gas chromatograph connected to an Agilent 5975C mass-selective detector (Agilent Technologies) was used. The analytical column used for GC/MS was a DB-WAX column for amines (60 m × 0.25 mm i.d., GL Sciences, Tokyo, Japan). Helium (99.99995%) was used as a carrier gas at a constant pressure mode (56.07 kPa). The injector (desorption) temperature was 250 °C. The SPME fiber was desorbed in the spitless mode, and the desorption time was 1 min. The oven temperature program ranged from 40 °C (4 min) to 240 °C at 6 °C/min. Electron ionization was used in the MS with a scan range of 35–300 m/z. The ion source temperature was maintained at 230 °C.

### Odorant-capturing property of the olfactory mucus and saliva

An odorant mixture solution (0.2 μL) containing Amb, Muscone, and PEA dissolved in ethanol was added to 20 μL of saline, BSA solution, olfactory mucus, denatured OC mucus, and saliva in a 2-mL headspace vial (Agilent Technologies). This was instantly sealed with a PTFE/silicone septum screw-cap. The headspace odorants were analyzed using SPME GC/MS as previously described. The GC/MS analytical column was a VF-WAXms column (30 m × 0.25 mm i.d., Agilent Technologies). The extracted ion chromatogram (EIC) peak area of odorants (m/z = 252 for Amb, 238 for Muscone, and 91 for PEA) was adjusted according to the ethanol EIC peak area (m/z = 45) among the samples. After extraction with SPME, the aqueous solution was diluted with water (80 μL), and ethyl acetate (100 μL) was added. The solution was vortexed to extract residual odorants in the organic layer. The organic layer was analyzed using GC/MS. The injected sample was 1 μL in the split mode (10:1).

### Enzymatic reactions with olfactory mucus, saliva, and CES-1

CES-1 inhibitors (BNPP and benzil) and recombinant CES-1 were purchased from TCI (Tokyo, Japan) and R&D Systems (Minneapolis, MN, USA), respectively. For enzymatic reactions, odorant mixtures (0.2 μL, 10 mM in ethanol) were added to 20 μL of saline, saliva, INM mucus, OC mucus, diluted samples (with saline), and CES-1 solutions (50 mM Tris–HCl, pH 7.5) with or without CES-1 inhibitors (aqueous solution for BNPP and dimethyl sulfoxide [DMSO] solution for benzil, 100 μM at final concentration). The solution was incubated at 37 ℃ for 1 h, then diluted with 80 μL of water. Ethyl acetate (100 μL) was added to the solution: the biphasic solution was vortexed to extract odorants and metabolites. The organic layer was collected and analyzed using GC/MS as previously described. Concentrations were determined using the area of the EIC peak. In the analysis of the structure–activity relationships with various acetates, the peak area of the ester **19** was almost unchanged across the experiments, and the corresponding alcohol was not detected. Therefore, the peak area among the experiments was adjusted using the peak area.

To determine the time-course of the enzymatic reaction of *p*CA, 1.6 μL *p*CA (10 mM in ethanol) was added to 160 μL of × 10 diluted OC mucus, CES-1 solution (0.57 μg/mL), and vehicles. Then, 20 μL of the reaction solution was collected at 0, 1, 5, 10, 20, 40, 60, and 120 min after the addition of *p*CA, incubated at 37 ℃, and subjected to GC/MS. To determine the Michaelis–Menten value, *p*CA was added to the diluted OC mucus or CES-1 solution, at final concentrations of 50, 100, 250, 500, and 1000 μM. The reaction solutions were incubated at 37 ℃ for 5 min (diluted OC mucus) or 40 min (CES-1 solution), and the reaction rates were determined by GC/MS analysis. The Michaelis–Menten values Vmax and Km were calculated using GraphPad Prism software (GraphPad Software, San Diego, CA, USA). *p*CA (final concentration = 100 μM) was used to determine the enzymatic reactivity of individual OC mucus samples (incubation time = 5 min).

### Sensory test to evaluate adaptation

*p*CA was prepared as a 0.1% mineral oil solution. The *p*-cresol concentration was 0.001% in distilled water. The odorant solutions (1 mL) were placed in 110-mL glass vials. Muscone was directly applied to sterilized cotton balls (1.0 mg) in a vial. The vials were left open to equilibrate the headspace. The number of participants with the OR9Q2 genotype was not determined, as no missense variants were reported. Eight participants were asked to smell *p*-cresol, and the perceived intensity of the odor was recorded on a 95-mm scale, marked from no odor to strong odor. They were then given one sample (water, *p*-cresol, muscone, or *p*CA) and were asked to inhale for 2 min. *p*-Cresol was presented again in a different vial, and participants were asked to rate the perceived intensity of the odor again. The intensity change was calculated as follows: before desensitization values were normalized as 100% and then compared with values after desensitization. The ranking was either a positive value (increased intensity of *p*-cresol after desensitization) or a negative value (decreased intensity of *p*-cresol after desensitization) to a minimum of − 100 (no perceived odor after desensitization). In each test, two separate trials with the same odorant were presented as Sample A. The participant rankings of *p*-cresol presented at separate times were compared to determine reliability. If the difference between the rankings was > 30%, the assessment was deemed unreliable and the intensity rankings were arbitrarily assigned. The results were then excluded from the analysis. Participants were given a 10-min break between each trial to reverse the effects of desensitization.

### CRE-regulated luciferase reporter gene assay

Dual-Glo™ luciferase assay (Promega, Madison, WI, USA) was performed as previously described^55^. Briefly, FLAG-Rho-tagged OR, cAMP response element (CRE)/luc2PpGL4.29, pRL-CMV, and RTP1S were added to Dulbecco’s Modified Eagle Medium (DMEM) with polyethylenimine Max (PEI-MAX, Polysciences, Warrington, PA, USA) for each well of a poly-d-lysine-coated 96-well plate (Corning) or poly-d-lysine-coated 384-well plate (Corning). After 15 min of incubation, the cell suspension was added to the transfection solution. After 24 h of transfection, the medium was removed, and the transfected cells were stimulated with an odorant solution diluted in DMEM.The 96- and 384-well plates were sealed and incubated at 37 °C for 3–4 h, and then luciferase reporter gene activities were measured. Odorant-induced activity was calculated as the CRE:luc ratio (luminescence intensity of firefly luciferase divided by that of Renilla luciferase) or fold-increase (Luc(N) divided by Luc(0)). Luc(N) was the CRE:luc ratio of a specific odorant-stimulated well, and Luc(0) was the CRE:luc ratio of a specific non-stimulated well. Data analysis was performed using Microsoft Excel or GraphPad Prism software.

### Calcium imaging for OR9Q2

The experiment was conducted as previously described^65^. Cells were seeded onto 35-mm glass-bottomed dishes (Iwaki Inc., Chiba, Japan) coated with poly-d-lysine. Then, cells were transfected with Rho-OR9Q2, Gα15, and RTP1S with PEI-MAX. After 24 h of incubation, Fura-2/AM-loaded cells were then washed with Ringer’s solution (140 mM NaCl, 3 mM KCl, 2 mM CaCl_2_, 1 mM MgCl_2_, 10 mM glucose, and 10 mM HEPES [pH 7.4]) and subjected to calcium imaging. A series of odorants in Ringer’s solution were applied sequentially to the cells using a peristaltic pump at a flow rate of 2.0 mL/min. Intracellular Ca^2+^ levels were detected as Fura-2/AM fluorescence at 510 nm by excitation at 340 or 380 nm using AQUA COSMOS (Hamamatsu Photonics, Shizuoka, Japan).

### Effects of medium volume on OR responses

The GloSensor™ cAMP Assay System (Promega, Madison, WI, USA) assessed the effect of medium volume on real-time OR responses to the vapor phase of odorants (Amb or *p*-cresol). HEK293T cells were transfected with 30 ng/well RTP1S plasmid, 67.5 ng/well OR9Q2 plasmid, or 30 ng/well cOR5A2 plasmid, and 67.5 ng/well 20F plasmid (Promega) on a 96-well black plate (Corning, Glendale, AZ, USA). At 24–40 h post-transfection, DMEM (Thermo Fisher Scientific) was replaced with 50 μL of CO_2_ independent medium (Thermo Fisher Scientific) containing 4% GloSensor cAMP reagent (Promega). After 2 h of equilibration at 37 ℃, the volume of the medium was adjusted from 0 to 50 μL in increments of 10 μL. Immediately thereafter, a 7-mm cotton ball (Osaki Medical, Nagoya, Japan) was placed above the medium in each well. The plate was loaded into a functional drug-screening system (FDSS)/µCELL (HAMAMATSU Photonics, Shizuoka, Japan). After 20 min of equilibration at 37 ℃, 150 μL odorant aqueous solution was automatically applied to each cotton ball at 50 μL/s. Cell luminescence was measured at 5-s intervals for 60 min. At each time point, a raw luminescence value from odorant-stimulated cells was subtracted by a value from non-stimulated cells. The ratio of the individual subtracted value to the maximum value in the 50 μL condition was defined as the normalized response (%).

### Ca^2+^ imaging for TRPM8

Calcium influx assays were performed using an FDSS/µCELL. The cells in which TRPM8 was stably expressed were obtained, as previously described^66^. The cells were seeded into poly d-lysine-coated 96-well plates (Corning) at 20,000 cells/well and cultured overnight. The cells were then incubated with Ringer’s solution, supplemented with 2 µM Fluo4-AM, 0.5 mM probenecid, and 0.01% Pluronic F-127, at 37 °C for 1 h. The cells were washed once and recovered with the assay buffer. Subsequently, the plates were inserted into the FDSS and the cells and test samples were preincubated for 5 min. The assay buffer was pre-warmed to 37 °C, and the assay was performed at 30 °C in the FDSS. Maximal [Ca^2+^]_i_ responses were measured as the peak fluorescence intensity ratio (peak fluorescence intensity/basal fluorescence intensity) and expressed as percentages of the response to 4 μM ionomycin.

## Supplementary Information

Supplementary Information 1.

Supplementary Table 1.

## Data Availability

All data discussed in the paper are available in the manuscript or the SI Appendix.
