# Supplementary material for: Functions of human olfactory mucus and age-dependent changes
Source: Sci Rep. 2023 Jan 18;13:971. doi: 10.1038/s41598-023-27937-1 (PMC9846672; doi:10.1038/s41598-023-27937-1)
Supplement: Supplementary file 1 — Supplementary Information 1. [file 41598_2023_27937_MOESM1_ESM.pdf]

Supplementary Information

Functions of human olfactory mucus and age-dependent changes

Shirai Tomohiro. et al.

Supplementary Table 1. individual-level data for olfactory mucus/saliva properties and perception (additional excel file)
Supplementary Table 2. Purchase source for odorants
Supplementary Table 3. ICP-MS measurement condition

Supplementary Fig. 1. Additional information for property of olfactory mucus.
Supplementary Fig. 2. Odorant-capturing capacity of OC mucus
Supplementary Fig. 3. Enzymatic reactivity of olfactory mucus.
Supplementary Fig. 4. ORs for *p*-cresol and *p*CA
Supplementary Fig. 5. Correlations of age and odor threshold scores for PEA and *tert*-butyl mercaptan (*t*BM)

Supplementary Table 2. Purchase source for odorants

Number in the article	Odorant name	Purchase source
**1**	*p*-Cresyl acetate (*p*CA)	Tokyo Chemical Industry Co. Ltd. (TCI, Japan)
**2**	*p*-Cresol	TCI
**3**	2-Phenylethyl acetate	TCI
**4**	Phenylethyl alcohol (PEA)	TCI
**5**	Anisyl acetate	TCI
**6**	Anisyl alcohol	TCI
**7**	Cinnmayl acetate	TCI
**8**	Cinnamyl alcohol	TCI
**9**	(-)-Menthyl acetate	TCI
**10**	*l*-Menthol	TCI
**11**	*cis*-3-Hexenyl acetate	TCI
**12**	*cis*-3-Hexenol	TCI
**13**	Citronellyl acetate	TCI
**14**	Citronellol	Fujifilm Wako (Japan)
**15**	*trans*-2-Hexenyl acetate	TCI
**16**	*trans*-2-Hexen-1-ol	TCI
**17**	Linalyl acetate	TCI
**18**	Linalool	TCI
**19**	α-Terpinyl acetate	TCI
**20**	α-Terpineol	TCI
**21**	Isobornyl acetate	TCI
**22**	Isoborneol	TCI
**23**	Benzaldehyde	TCI
**24**	Benzylalcohol	TCI
**25**	Octanal	TCI
**26**	Octanol	TCI
**27**	Octanoic acid	TCI
**28**	2'-Methoxyacetophenone	TCI
**29**	2'-Hydroxyacetophenone	TCI
**30**	Acetophenone	TCI
**31**	Methyl salicylate	TCI
none	Ambrettolide (Amb)	Sigma-Aldrich (USA)
none	*tert*-Butyl mercaptan (*t*BM)	TCI
none	*l*-Menthone	TCI
none	Muscone	MP BioMedical (USA)

Supplementary Table 3. ICP-MS measurement condition

ICP-MS measurement conditions		
Instrument	ThermoFisherScientific iCAP Qs	
	Method	Quadrupole type
Analytical elements		(^{23}Na, ^{24}Mg, ^{27}Al, ^{39}K, ^{40}Ca, ^{52}Cr, ^{55}Mn, ^{56}Fe, ^{60}Ni, ^{59}Co,^{63}Cu,) ^{66}Zn
Plasma conditions and parameters	RF power	1550 W (cold plasma : 700W)
	Sampling depth	5 mm (cold plasma : 10mm)
	Plasma gas flow	14 L/min
	Carrier gas flow	0.8 L/min
	Additional gas(20%O_2/Ar) flow	20% (cold plasma : 30%)
	Nebulizer gas flow	0.65 L/min
	Collision gas (He) flow	4.9 mL/min (cold plasma : −)
	Collision reaction gas (1%NH_3-He) flow	— (cold plasma : 8.8 mL/min)
	Interface	Pt skimmer, Pt sample
	Injector	1mmΦ, quartz
	Nebulizer type	MicroFlow PFA-100 Coaxial type (self-aspirating)
	Spraychamber	Quartz cyclonic
	Peltier Temperature	4℃
Integration time	0.1 sec	
Number of measurements	3	
Number of calibration curve points	9	

The measurement conditions in parentheses ( ) were applied to the measurement of the elements in parentheses ( ).

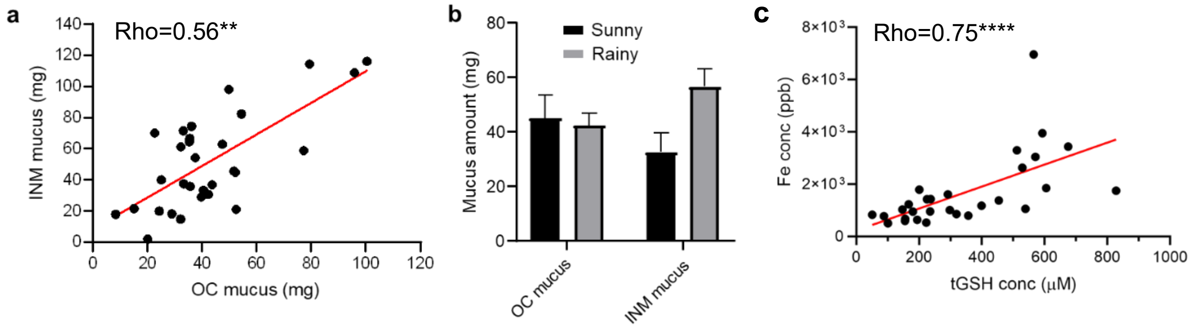

Supplementary Fig. 1 Additional information for property of olfactory mucus.

a Spearman's correlation of OC mucus amount and INM mucus amount (Rho=0.56, **$p < 0.01$, n=30). **b** OC mucus amount and INM mucus amount on a sunny day or rainy days. n=6 for sunny group, n=24 for rainy group. Mean±SE. **c** Spearman's correlation of tGSH and Fe concentration in OC mucus (Rho=0.75, ****$p < 0.0001$, n=29).

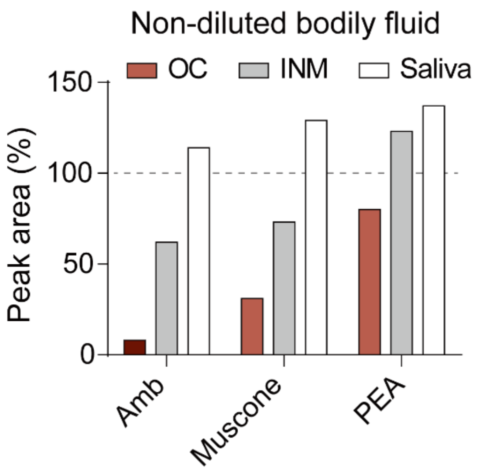

Supplementary Fig. 2 Odorant-capturing capacity of OC mucus

Relative headspace concentration of odorants emitted from OC mucus, INM mucus, or saliva. Each bodily fluid was tested as a mixture of equal amounts from all participants. Odorants were added with ethanol for a final concentration of 500 μM (1% EtOH). The peak area of each odorant without bodily fluid was set to 100% after normalization with the peak area of ethanol.

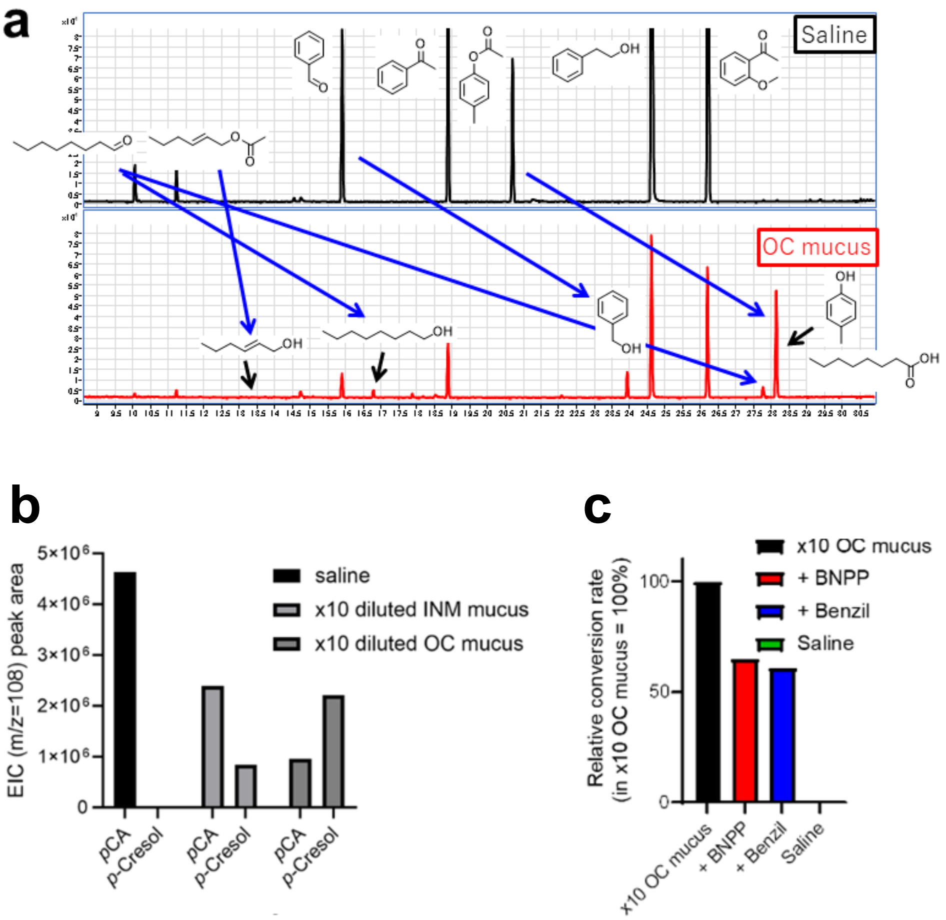

Supplementary Fig. 3 Enzymatic reactivity of olfactory mucus.

a An example of experiments which evaluates enzymatic reactivity of bodily fluids. Seven odorants (Structures were shown in the upper figure, “saline”) were incubated in saline or OC mucus. Ethyl acetate extract of the reactant was analyzed using GC/MS. Total ion chromatograms after mixing with saline and OC mucus are shown. **b** OC mucus showed higher esterase activity to *p*CA than INM mucus even when tested in 10-fold dilution. Peak area of *p*CA and *p*-cresol in elected ion chromatogram (m/z = 108) of GC/MS after incubation with saline, x10 diluted OC mucus, and x10 diluted INM mucus. **c** Effect of CES1 inhibitors BNPP and benzil for *p*CA metabolism in x10 OC mucus.

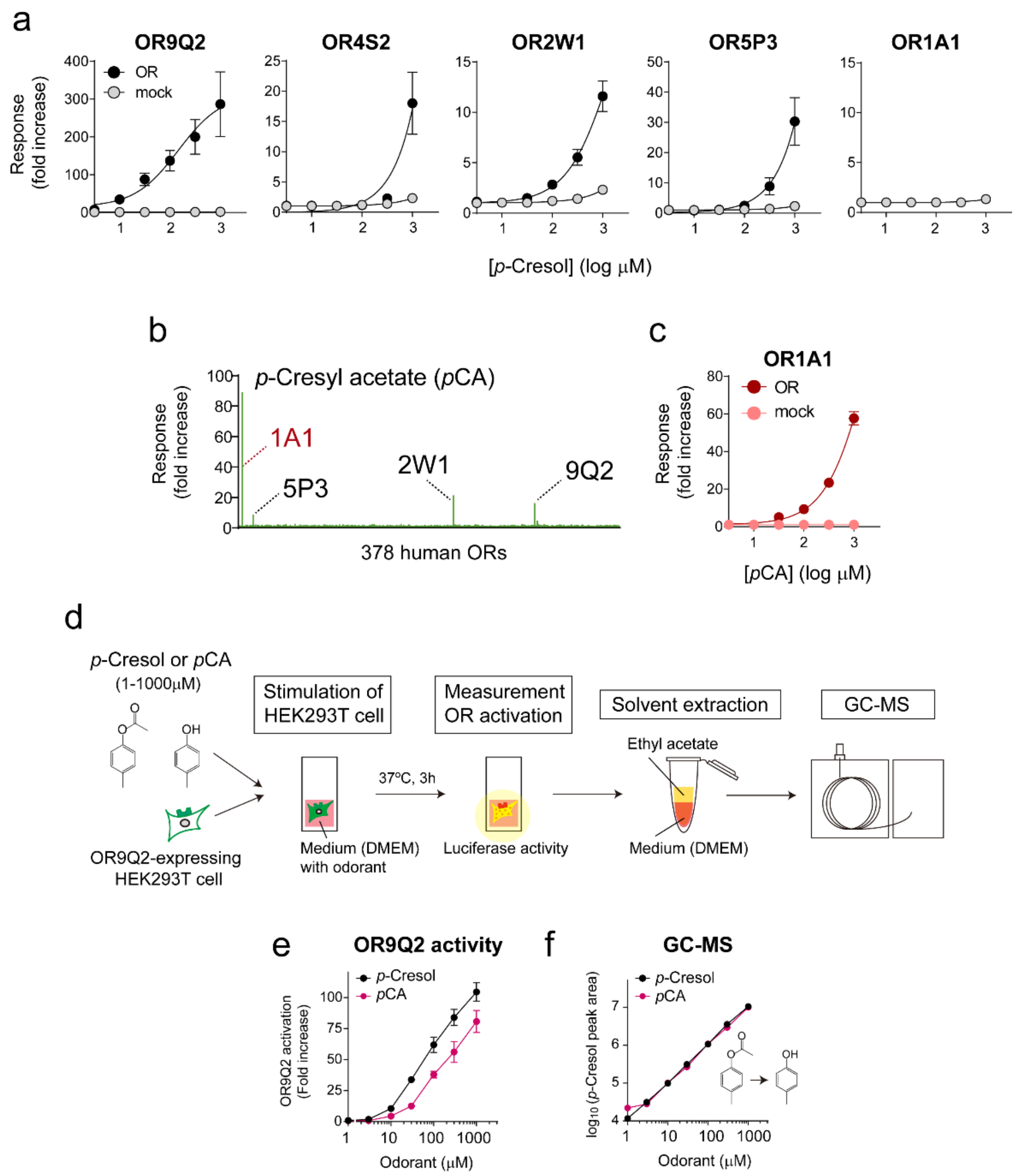

Supplementary Fig. 4 ORs for *p*-cresol and *p*CA

a Responses of HEK293T cells transfected with each OR was monitored using CRE-luciferase reporter gene assay. Cells transfected with an empty vector were also assayed (mock, grey circle). Data were shown as mean±S.E. from three independent experiments. **b** Screening of human ORs for *p*CA. Each of the 378 ORs listed along the x-axis was expressed in HEK293T cells and stimulated with *p*CA (1 mM). **c** Dose-response curve of OR1A1 activation with *p*CA. **d** Experimental procedure for the quantification of enzymatic conversion of *p*CA by carboxylic ester hydrolases activity of HEK293T cells. After measurement of luciferase activity, odorants applied to OR-expressing HEK293T cells were extracted from medium with an organic solvent and analyzed by GC/MS. **e** Dose-response curve of OR9Q2 activity with *p*CA or *p*-cresol. **f** GC-MS peak area (*m/z* 108) of *p*-cresol. *p*-cresol was detected from *p*CA-stimulated medium after measurement of luciferase activity, while *p*CA was not detected.

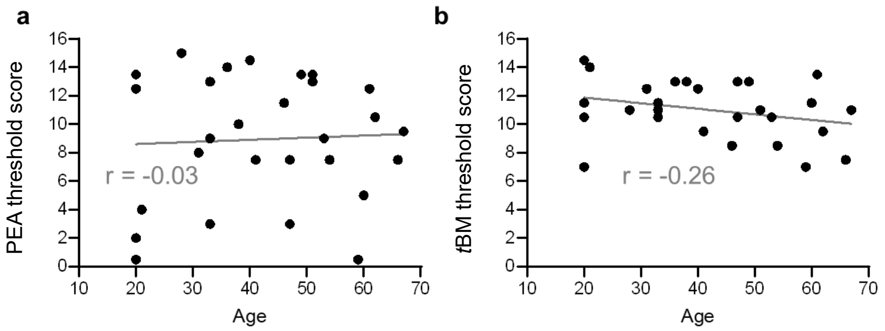

Supplementary Fig. 5 Correlations of age and odor threshold scores for PEA and *tert*-butyl mercaptan (*t*BM)

a,**b** Correlation of age and odor threshold score for PEA (a) and *t*BM (b)**.** Age-dependent score reduction was not observed. Spearman's correlations were not significant ($p > 0.05$, n=28).
